# Probing disorder in pyrochlore oxides using *in situ* synchrotron diffraction from levitated solids–A thermodynamic perspective

**DOI:** 10.1038/s41598-018-28877-x

**Published:** 2018-07-13

**Authors:** Pardha S. Maram, Sergey V. Ushakov, Richard J. K. Weber, Chris J. Benmore, Alexandra Navrotsky

**Affiliations:** 1 Peter A. Rock Thermochemistry Laboratory and NEAT ORU, University of California Davis, One Shields Avenue, 4415 Chemistry Annex, Davis, California, 95616 USA; 2 X-ray Science Division, Advanced Photon Source, Argonne National Laboratory, 9700S. Cass Avenue, Lemont, Illinois 60439 USA; 3 Materials Development, Inc., 3090 Daniels Court, Arlington Heights, Illinois 60004 USA

## Abstract

Pyrochlore, an ordered derivative of the defect fluorite structure, shows complex disordering behavior as a function of composition, temperature, pressure, and radiation damage. We propose a thermodynamic model to calculate the disordering enthalpies for several RE_2_Zr_2_O_7_ (RE = Sm, Eu, Gd) pyrochlores from experimental site distribution data obtained by *in situ* high temperature synchrotron X-ray diffraction. Site occupancies show a gradual increase in disorder on both cation and anion sublattices with increasing temperature and even greater disorder is achieved close to the phase transition to defect fluorite. The enthalpy associated with cation disorder depends on the radius of the rare earth ion, while the enthalpy of oxygen disordering is relatively constant for different compositions. The experimental data support trends predicted by *ab initio* calculations, but the obtained enthalpies of disordering are less endothermic than the predicted values. Thermal expansion coefficients are in the range (8.6–10.8) × 10^−6^ K^−1^. These new experimental determinations of defect formation energies are important for understanding the stability of pyrochlore oxides and their disordering mechanisms, which are essential in the context of their potential applications in nuclear waste management and other technologies.

## Introduction

Structural disorder of A_2_B_2_O_7_ pyrochlores (*PY*) is a major factor determining their physical properties and stability. Many undergo an order–disorder transformation at high temperatures, high pressures and under grinding or ion irradiation where the *PY* superstructure is lost, leaving long-range defect fluorite-type (*DF*) disorder^1–5^. Their functional properties are very sensitive to the degree of disorder^6–8^, which appears to be governed by radius ratio rules^9^. Despite extensive technological applications as fast ionic conductors, thermal barrier coatings, and ceramics for the immobilization of radioactive waste^4,10–13^, our understanding of their disordering mechanism and defect formation energies is still incomplete. Recently, Shamblin *et al*. pointed out that the structure of disordered *PY* produced by radiation damage is more complex than previously thought and may involve domains of a weberite-like structure with an intermediate degree of order^14–16^. The order–disorder transition from *PY* to *DF* is a rare example of simultaneous disordering in both anion and cation sublattices^17^. The most important defects that form in *PY* are the cation antisite (CA) and anion Frenkel pair (AFP), with their formation driving the phase transitions^9,18^. Several authors have investigated the relationship between the structural stability of *PY* oxides and the defect formation energies (DFE) using computational techniques, and the results have been widely used to interpret the experimental findings^19^.

Sickafus *et al*. presented contour plots of DFE across a wide variety of *PY* oxides using force–field atomistic simulations (FFA)^3,20,21^. Panero *et al*. applied DFT methods to the DFE in Y_2_(Ti, Sn, Zr)_2_O_7_, and the results indicate that the CA pair has lower energy (0–2 eV) than the AFP (4–11 eV). They also found that the stannates like Y_2_Sn_2_O_7_ have larger DFE than titanates and zirconates because of the greater covalent character of the Sn–O bond compared to Ti–O and Zr–O^22,23^. Chartier *et al*. simulated radiation damage cascades in Ti-doped La_2_Zr_2_O_7_ and found a significant difference in Zr–La and Ti–La CA DFE, supporting the observation that titanate *PYs* may be unfavorable for radioactive waste immobilization as radiation damage causes amorphization rather than disordering^24^. All of the reported computational studies support experimental correlations that link observed *PY* – *DF* transformations with simple radius ratio (r_A_/r_B_) rules^9^. However, the predicted energies (at least 2–3 eV)^20,22,24^ appear to be too large for significant disordering to occur prior to melting. On the other hand, recent DFT studies on select *PY* oxides show that these energies are substantially smaller than those predicted with FFA methods^25^. It is clear that the computational studies need comparison with experimental determinations of DFE. Our goal is to develop an *in situ* experimental method for quantitative determination of site distributions from which DFE can be calculated by a thermodynamic model. *In situ* structural studies are essential because the high temperatures involved in equilibrium disordering make it questionable whether the disordering can be preserved on “quenching” the sample to ambient conditions. Indeed, numerous authors have studied functional properties of *PY* oxides with different degrees of disorder made by quenching from high temperatures, but they achieved very low percentages of disorder (5–22%) and did not reach the fully disordered state^26–28^.

The disordering in *PY* oxides may be viewed as an equilibrium reflecting the balance of a positive enthalpy of interchange on cation and on anion sublattices and a positive configurational entropy of disordering. Such gradual disordering in spinels has been described by a simple thermodynamic model, treating cation distribution as a chemical equilibrium at a given temperature^29^. The cation distribution as a function of temperature can be used to calculate the appropriate interchange enthalpies for both CA and AFP. In this study, a similar thermodynamic model is applied to the disordering in *PY* oxides, with independent reactions representing CA and AFP disorder. For the first time, we have adopted a high temperature *in situ* diffraction technique to induce equilibrium cation and anion disorder within the *PY* samples by thermal treatment close to the melting point using aerodynamic levitation and laser heating. The site occupancies (atomic sublattice disorder), lattice constants and positional parameters were refined at a number of temperatures by Rietveld structure analysis, and the obtained site occupancies were then subjected to thermodynamic analysis.

## Results and Discussion

### Composition and thermal expansion

Chemical analysis of the melt-quenched spheroids by microprobe showed stoichiometric composition within experimental error, see Table 1. The room temperature synchrotron X-ray diffraction pattern of all the melt-quenched compositions, showed intense and sharp patterns, and no impurity phases could be detected (Figure S1 in SI). All the compositions showed *DF*-based diffraction peaks, (222), (400), (440) and (622). Also, superlattice reflections, (111), (311), (331) and (511) are seen in La_2_Hf_2_O_7_, Nd_2_Hf_2_O_7_, Sm_2_Hf_2_O_7_, Nd_2_Zr_2_O_7_, and Sm_2_Zr_2_O_7_, indicating the doubling of the cubic unit cell. The cation radius ratio, 1.46 ≤ r_A/_r_B_ ≤ 1.80 governs the formation, and structural stability of the oxide *PYs*^9^. For the rare earth zirconate series, Gd_2_Zr_2_O_7_ (r_Gd3+_/r_Zr4+_ = 1.46) is considered the boundary between *PY* and *DF* structures^9^. The absence of superlattice reflections in the melt-quenched Eu_2_Zr_2_O_7_ and Gd_2_Zr_2_O_7_ samples indicate the *DF* structure^30^. Thus, during quenching from the melt, compositions with r_A_/r_B_ at and slightly above 1.46 formed *DF* structures. The calculated room temperature lattice parameters are given in Table 1. A systematic decrease in lattice constant is observed as the ionic radius of the lanthanide decreases.

**Table 1:** Chemical composition, room temperature lattice parameter and melting temperatures for all the studied *PY* oxides after quenching from the melt.

Composition by Microprobe analysis^†^	N^‡^	*Lattice constant, Å	Melting temp. from cooling traces, T(K)	^§^Melting temp. from *in situ* diffraction Tv(K)
La_1.97(2)_Hf_2.02(1)_O_7_	10	10.7916(1)	2633 ± 10	2673
Nd_1.94(4)_Hf_2.05(3)_O_7_	7	10.6455(1)	2706 ± 20	2773
Sm_2.07(6)_Hf_1.95(5)_O_7_	10	10.5840(1)	2843 ± 30	2873
Nd_1.97(12)_Zr_2.02(9)_O_7_	9	10.6607(1)	2650 ± 50	2673
Sm_1.98(13)_Zr_2.02(10)_O_7_	10	10.5965(2)	2836 ± 14	2823
Eu_2.01(8)_Zr_1.99(10)_O_7_	10	10.5489(1)^#^	2343 ± 10	—
Gd_1.91(5)_Zr_2.07(5)_O_7_	13	10.5258(1)^#^	—	—

^**†**^Error in parenthesis is a standard deviation in the last digit. ^**‡**^N = Number of data points used to calculate the average composition of a respective phase. *Room temperature lattice parameter from synchrotron diffraction. No recalescence peak observed for Gd_2_Zr_2_O_7_ in cooling profile. ^#^Melt quench phase is defect fluorite and the lattice parameter is doubled for direct comparison. ^§^Melting temperatures corrected with reference to the thermal arrest observed in cooling trace profile of the respective composition.

**Figure 1:**
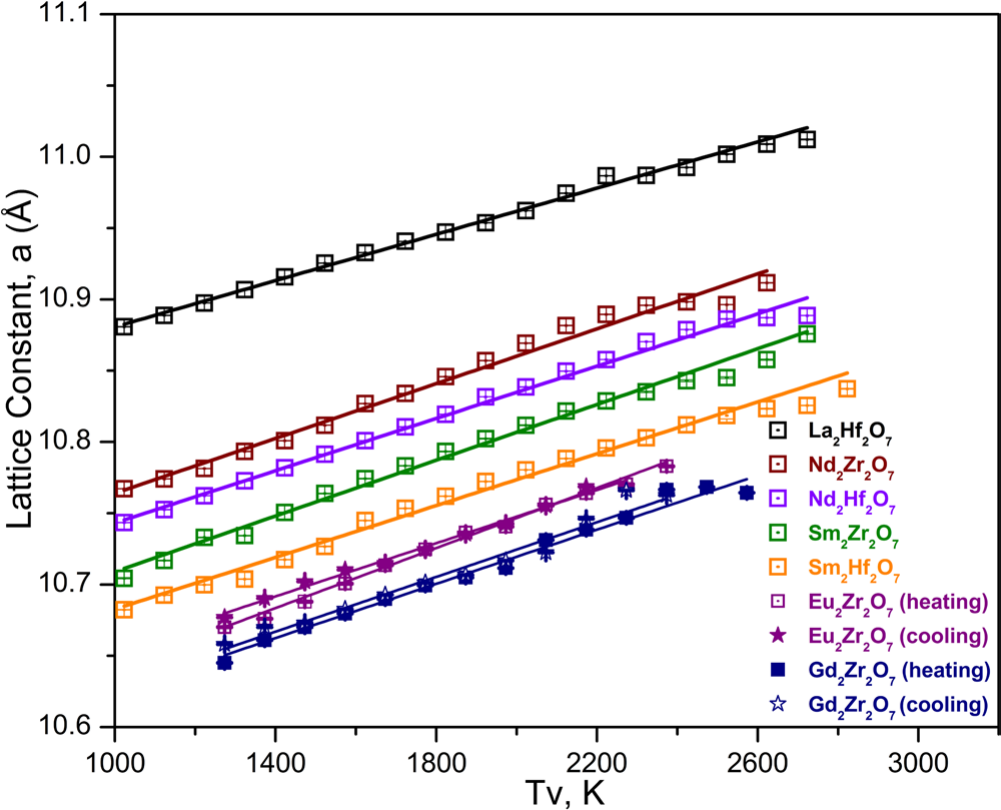
The change in lattice constant versus diffracted volume temperature (T_v_) for all the studied pyrochlore compositions. For Eu_2_Zr_2_O_7_ and Gd_2_Zr_2_O_7_, the plot is between lattice constant and actual measured temperature (T, K). The error bars are smaller than the symbol size.

For all the compositions tested, the unit cell size changes smoothly as a function of temperature till melting. No anomalous lattice constant behavior is detected at high temperature, confirming that the applied temperature corrections are reasonable. For comparison, the temperature range selected for linear regression analysis was fixed to 1123–2323 K. The variation of thermal expansion coefficients (TEC) vs. temperature is within reported uncertainty from the linear regression fit, so a temperature independent TEC is supported. Some general trends can be noticed from Table 2 and Fig. 1, TEC increases as the size of the lanthanide ion decreases, and the zirconate series has higher TEC than the hafnate series. TEC of studied compositions varies from (8.6 to 10.8) × 10^−6^ K^−1^, the lowest TEC being observed for La_2_Hf_2_O_7_. The highest TEC is seen for Sm_2_Zr_2_O_7_ and Gd_2_Zr_2_O_7_ = (10.8 ± 0.5) × 10^−6^ K^−1^ and (10.6 ± 0.3) × 10^−6^ K^−1^. The TEC values within the studied temperature range, 1123–2373 K, measured by the levitation method, agree well with previously reported values of 8–11 × 10^−6^ K^−1^ in the 298–1473 K temperature range^31^.

**Table 2:** Coefficients of Lattice Parameter Equations a = a_0_(1 + LT), T in K (1123 to 2323 K) and Mean Linear Thermal Expansion Coefficient (α).

Composition (phase)	a_o_ (Å)	^†^α x10^−6^ (1/K)	R^2^	^‡^α x10^−6^ (1/K)
La_1.97(2)_Hf_2.02(1)_O_7_	10.777(5)	8.78(30)	0.985	8.71(52)
Nd_1.94(4)_Hf_2.05(3)_O_7_	10.628(5)	9.99(29)	0.989	9.88(52)
Sm_2.07(6)_Hf_1.95(5)_O_7_	10.564(3)	10.22(31)	0.988	10.11(34)
Nd_1.97(12)_Zr_2.02(9)_O_7_	10.639(5)	10.78(28)	0.991	10.66(64)
Sm_1.98(13)_Zr_2.02(10)_O_7_	10.579(7)	11.06(40)	0.983	10.93(42)
Eu_2.01(8)_Zr_1.99(10)_O_7_				
Heating (DF-P-DF)	10.524(5)	10.62(25)	0.993	10.50(30)
Cooling (DF-P)	10.529(8)	10.46(44)	0.980	10.32(15)
Gd_1.91(5)_Zr_2.07(5)_O_7_				
Heating (DF-P-DF)	10.499(6)	10.61(31)	0.990	10.47(23)
Cooling (DF-P)	10.500(5)	10.79(31)	0.990	10.64(18)

a_0_ correspond to cell parameter at 298 K, L is thermal expansion coefficient ^**†**^Corresponds to thermal expansion coefficient at 298 K; ^**‡**^Calculated as α = (a_(2323K)_ − a_(1123 K)_)/a_(1123K)_/1200; number in parenthesis represents the decimal point variation.

### Pyrochlore structure as a function of temperature

During heating, the *PY* La_2_Hf_2_O_7_ and Nd_2_Hf_2_O_7_ persist to the melting temperatures, 2673 and 2773 K, respectively (Figures S2 & S3 in SI). In Nd_2_Zr_2_O_7_, all the superlattice reflections are present to 2523 K, but with further increase in temperature, the (311) reflection disappears, but (111), (331), and (511) reflections persist to melting at 2623 K (Figure S4 in SI). It is known that Nd_2_Zr_2_O_7_ shows a *PY* to *DF* phase transition, with a reported transition temperature between 2493 and 2573 K^1,32^. In this study, the (311) reflection disappears at 2423 K, within the reported temperature range, confirming the existence of the phase transition. However, the presence of residual superlattice reflections till melting may be due to the presence of small *PY* domains within the *DF* matrix^33^. Also, inhomogeneity in specimen temperature could cause the observed residual superlattice reflections. For Sm_2_Hf_2_O_7_, the reflections of the *PY* structure intensify up to 2123 K, but further increase in temperature weakens the superlattice reflections, that then disappear completely, indicating transformation to *DF* just before melting at 2823 K (Figure S5 in SI).

**Figure 2:**
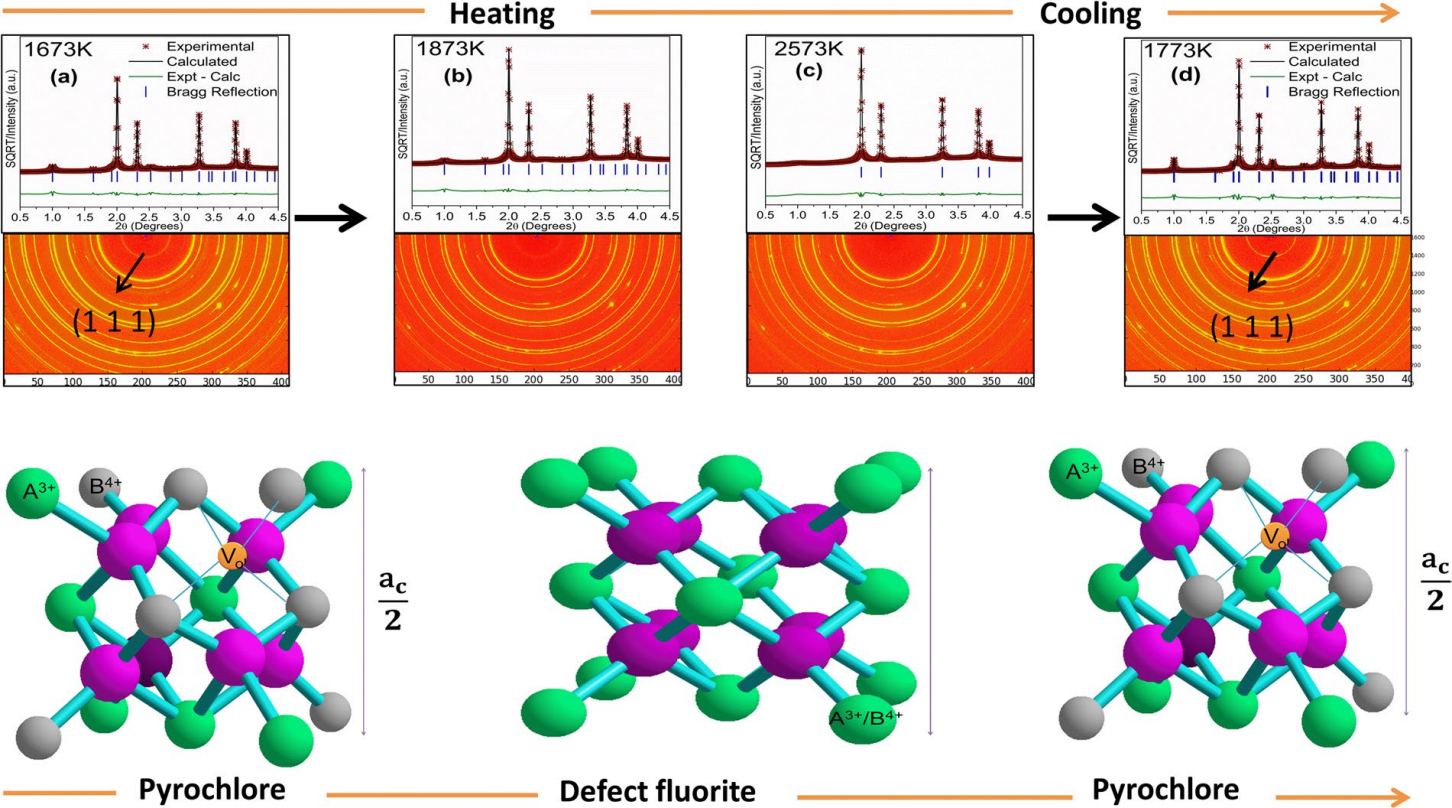
(**a**–**d**) Synchrotron X-ray diffraction images and the corresponding Rietveld structure refinement plots for the melt quench Gd_2_Zr_2_O_7_ at different temperatures. The inner pyrochlore ring (111) fades during heating at 673–1873K, completely disappears at 2573 K (**a**–**c**), then reappears during cooling (**d**), confirming reversible order–disorder phase transformation.

For Sm_2_Zr_2_O_7_, the superlattice reflections disappear, indicating transformation to *DF* at 2323 K, and melting occurs at 2823 K (Figure S6 in SI). For Eu_2_Zr_2_O_7_, previously we reported a reversible *PY–DF* phase transition at 2173 K during heating and 2073 K during cooling (Figure S7 in SI)^5^. Similarly, Gd_2_Zr_2_O_7_ shows a reversible *PY*–*DF* phase transformation during heating and cooling in the range 1773–1873 K (Figure S8 in SI). Synchrotron diffraction patterns of all the compositions as a function of temperature and the results of profile fitting for La_2_Hf_2_O_7_, Nd_2_Zr_2_O_7_, Nd_2_Hf_2_O_7_, and Sm_2_Hf_2_O_7_ are given in Figures (S2–S8**)** and Table S1 in the SI. Figure 2(a–d) shows the 2D synchrotron diffraction images tracking the structure change as a function of temperature and the corresponding Rietveld refinement plots. Since the sample is initially in the metastable *DF* phase (beads made by melt quench), during heating superlattice reflections (111), start to appear around 1473 K and become significant at 1673 K (Fig. 2a). Further increase in temperature to 1873 K shows minor superlattice reflections (Fig. 2b), while above this temperature no (111) reflection is visible (Fig. 2C), confirming the phase transition to *DF*. Similar behavior is also observed during cooling; from 2573 to 1773 K there is a (111) superlattice reflection indicative of *PY* structure (Fig. 2d). Thus, a stepwise first-order (with some hysteresis) reversible phase transformation from ordered (*PY*) to disordered (*DF)* is captured for Gd_2_Zr_2_O_7_.

**Figure 3:**
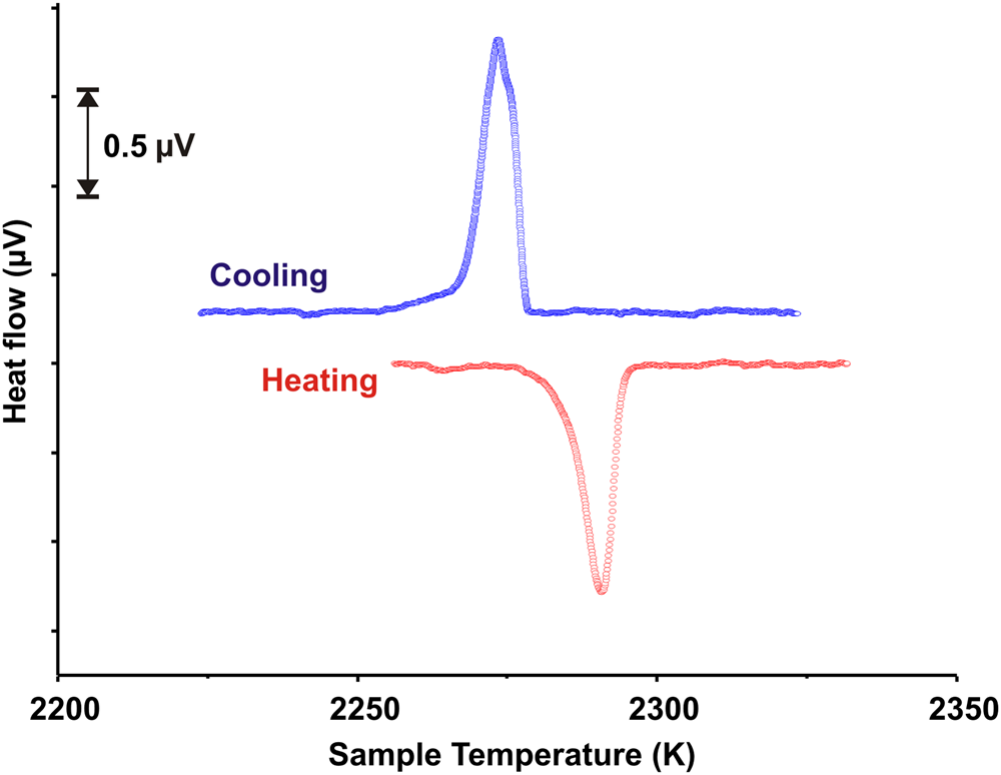
Ultra-high-temperature differential thermal analysis performed on Sm_2_Zr_2_O_7_. The given heating and cooling curves are shown after correcting the sample temperature using Al_2_O_3_ melting. Heating rate = 20 degrees/min.

We conducted high-temperature differential thermal analysis (DTA) on Sm_2_Zr_2_O_7_, Eu_2_Zr_2_O_7_, and Gd_2_Zr_2_O_7_. Distinct endothermic (heating) and exothermic (cooling) peaks are observed for Sm_2_Zr_2_O_7_, and Eu_2_Zr_2_O_7_, but no peaks are observed in Gd_2_Zr_2_O_7_. For example, Fig. 3 shows the heating/cooling traces observed for Sm_2_Zr_2_O_7_. The DTA traces indicate a phase transformation at 2282 ± 10 K for Sm_2_Zr_2_O_7_ and 2100 ± 18 K for Eu_2_Zr_2_O_7_. The transition enthalpies for Sm_2_Zr_2_O_7_ and Eu_2_Zr_2_O_7_ were obtained by integrating the high-temperature DTA signal using the melting enthalpy of corundum as a calibration. The measured phase transition enthalpies are 11 ± 2 kJ mol^−1^ on heating and −14 ± 2 kJ.mol^−1^ on cooling for Sm_2_Zr_2_O_7_ and 7.4 ± 0.4 kJ mol^−1^ on heating and −8.3 ± 1.0 kJ.mol^−1^ on cooling for Eu_2_Zr_2_O_7_. The newly measured transition temperatures from *in situ* synchrotron diffraction are 2323 K for Sm_2_Zr_2_O_7_ and 2123 K for Eu_2_Zr_2_O_7_, corroborating the DTA studies.

A few reports exist on the high-temperature *PY*–*DF* transformations and the existing data show discrepancies. The first report was by Michel *et al*. on Ln_2_Zr_2_O_7_ (Ln = La, Nd, Sm, Gd)^1^. The reported phase transformation temperatures are 2573 K for Nd_2_Zr_2_O_7_, 2273 K for Sm_2_Zr_2_O_7_, 1803 K for Gd_2_Zr_2_O_7_ and 2673 K for Gd_2_Hf_2_O_7_. Zoz *et al*. reported the transformation at 1993–2053 K in Eu_2_Zr_2_O_7_^34^. Fabrichnaya *et al*. corrected the *PY*–*DF* transition in Eu_2_Zr_2_O_7_ to 2128 K^35^. The phase transition temperatures obtained by *in situ* diffraction and DTA generally show good agreement with literature reports (see Table 3). These observations confirm that aerodynamic levitation combined with *in situ* diffraction is a very reliable technique to study the phase equilibria of high-temperature ceramics.

**Table 3:** The *PY- DF* phase transition temperature and entropy change at T_c_ (critical transition temp).

	Sm_2_Zr_2_O_7_	Eu_2_Zr_2_O_7_	Gd_2_Zr_2_O_7_
T_c_, K (Literature)	2273^1^	2128^31^	1803^1^
T_c_, K (*in situ*)	2323	2073	1773
T_c_, K (DTA)			—
Heating	2285 ± 10	2113 ± 18	
Cooling	2278 ± 10	2087 ± 12	
*ΔH*_*trans*_, kJ.mol^−1^ (DTA)			—
Heating	11.0 ± 2.0	7.4 ± 0.4	
Cooling	−14.0 ± 2.0	−8.3 ± 1.0	
*ΔS*, J.mol^−1^.K^−1^ (DTA)			
Heating	4.8 ± 0.9	3.5 ± 0.2	—
Cooling	−6.2 ± 0.9	−4.0 ± 0.5	
*ΔS*_*conf*_, J.mol^−1^.K^−1^ (*site distribution*)			
Heating	6.9 ± 0.8	7.4 ± 0.5	—
Cooling	—	–6.1 ± 0.4	−5.9 ± 0.7

The given phase transformation temperature from *in situ* are average during heating and cooling. For Gd_2_Zr_2_O_7_ no heat signal observed in DTA experiment. The *ΔS*_*Conf*_ are calculated right below the transition temperature to that of completely disordered *DF*.

**Figure 4:**
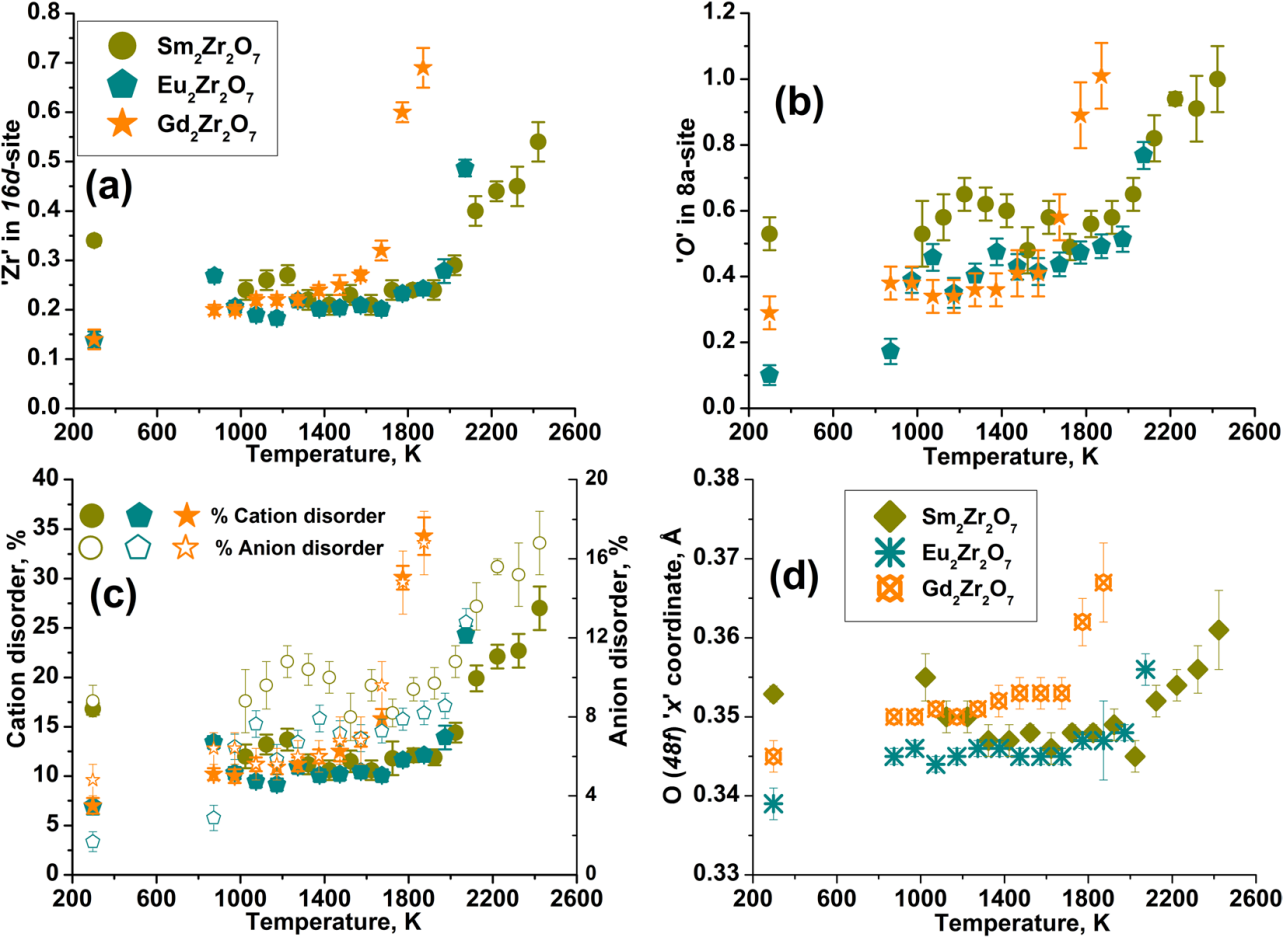
Change in (**a**) antisite cation occupancies, (**b**) anion Frenkel occupancy, (**c**) percentage of cation and anion disorder and (**d**) change in *48f* oxygen *‘x’* positional parameter as a function of temperature for Sm_2_Zr_2_O_7_, Eu_2_Zr_2_O_7_ and Gd_2_Zr_2_O_7_. In the case of Sm_2_Zr_2_O_7_, the temperatures are corrected to diffracted volume (T_v_). The Tv was calculated based on the observed difference in melting temperature from cooling trace and *in situ* diffraction.

Figure 4(a,b) presents the temperature dependence of antisite cationic and oxygen Frenkel occupancies of Sm_2_Zr_2_O_7_ (heating), Eu_2_Zr_2_O_7_ (cooling)_,_ and Gd_2_Zr_2_O_7_ (cooling). At lower temperature, the antisite occupancies change smoothly till 2023 K, 1873 K and 1573 K for Sm_2_Zr_2_O_7_, Eu_2_Zr_2_O_7_, and Gd_2_Zr_2_O_7_, respectively. Further increase in temperature shows an abrupt change in antisite occupancies indicating the phase transition to *DF*. In Eu_2_Zr_2_O_7_ (cooling), and Gd_2_Zr_2_O_7_ (cooling), as temperature decreases, disordering decreases and the structure becomes closer to the ideal *PY*. Disordering in both cation/ and anion sublattices is inevitable, and intrinsic to *PY* oxides. For example, it was reported that the as made stoichiometric *PYs* show disorder in the range of 5 to 10% at room temperature^26,36^. Shlyakhtina *et al*. observed cation antisite disorder of approximately 8, 5, and 22% for Sm_2_Zr_2_O_7_, Eu_2_Zr_2_O_7_, and Gd_2_Zr_2_O_7_, respectively^36^. Zhang *et al*. attempted to synthesize Gd_2_Zr_2_O_7_ samples with different degrees of the disorder by isothermal annealing at 1100–1500 °C. However, a perfectly ordered Gd_2_Zr_2_O_7_ *PY* structure was never attained even for extended periods of heat treatment. In Gd_2_Zr_2_O_7_, cation disorder of 36.3% was reported in a sample annealed at 1550 °C for 24 hours^37^.

From the current study, the percentages of cationic and anionic disorder as a function of temperature are given in Fig. 4(c). Gd_2_Zr_2_O_7_ shows the greatest cationic and anionic disorder, 34.3 ± 1.9% and 16.8 ± 1.6%, respectively. It is well-known that heavier lanthanides do not form ordered zirconates with *PY* structure and Gd is considered the borderline lanthanide between *PY* and *DF* structures^9^, so its greater disordering is not surprising. Direct comparison of refinements is not fully appropriate since our study includes Rietveld refinement of both cation and anion disorder whereas the majority of published results refined only cation antisite disorder^26,36,37^. However, quenching *PY* samples such that they would retain different equilibrium degrees of disorder may be difficult or even impossible, particularly for lighter lanthanides which strongly prefer ordered structures. In contrast, aerodynamic levitation and laser heating in combination with *in situ* synchrotron diffraction is a versatile technique to obtain the actual degree of disorder under high temperature conditions. Previously, we have shown the systematic behavior of cation and anion disorder in Eu_2_Zr_2_O_7_ during heating and cooling using this methodology^5^.

### Change of ‘x’ (*48f)* as a function of temperature

Disordering as a function of temperature can also be observed by monitoring the changes in the *48f* oxygen ‘*x’* positional parameter. In a large number of *PYs*, the ‘*x*’ parameter lies well below 0.375^9^. In the present study, *48f* oxygen ‘*x*’ parameters for Sm_2_Zr_2_O_7_, Eu_2_Zr_2_O_7_ and Gd_2_Zr_2_O_7_ are 0.346 (1), 0.339(2) and 0.345(2), respectively at room temperature. For Eu_2_Zr_2_O_7_ and Gd_2_Zr_2_O_7_, since the initial melt-quench phases are *DF*, the given ‘*x*’ values are after quenching from the melt. As the *48f* oxygen positional ‘*x*’ parameter changes smoothly with temperature, until reaching the *PY*–*DF* phase boundary, further increase in temperature abruptly raises the value toward 0.375 (see Fig. 4d). In Sm_2_Zr_2_O_7_, ‘*x*’ is 0.355(3) at 1123 K, showing slightly higher values as it has more disorder due to melt quench, and ‘*x*’ is lowered to 0.345(2) at 2273 K, indicating the change toward the ideal *PY* structure. Increasing the temperature to 2373 K raises ‘*x*’ to 0.352(2), and ‘*x*’ reaches a maximum of 0.361(5) at 2523 K close to the value for *DF* (0.375). In Gd_2_Zr_2_O_7_, during cooling from 2373 K to room temperature, the *PY* phase appears at 1873 K, and ‘*x*’ decreases from 0.367(5) to 0.345(2), indicating change toward the ideal *PY* structure. Previously, we have reported the changes in ‘*x*’ vs. temperature for Eu_2_Zr_2_O_7_^5^, and the trend correlates well with the current study. The change in lattice constant, refined occupancies, *48f* oxygen ‘*x*’ positional parameter, % disorder (both cation and anion), and agreement factors (R-factors namely Bragg R, R_f_-factor, and Chi2) during heating and cooling as a function of temperature are documented in Tables S2, S3 and S4 in SI.

## Thermodynamic Model of Disordering

Any high temperature equilibrium phase transactions involving order–disorder reflects a balance between the enthalpy of disordering and the configurational entropy created by disorder on crystallographic sites on the available sublattices. The equilibrium disordering can occur gradually with temperature, representing a second order or more complex transition, or it can occur sharply at one temperature (sometimes with hysteresis), representing a first order transition. In real systems, a transition can show complex behavior, with gradual disordering over a temperature range culminating in a first order transition, or with some short-range order persisting in the high temperature nominally disordered phase. A first-order transformation is accompanied by an abrupt change in enthalpy and entropy. The Gibbs free energy difference between the two phases is

1 $${\rm{\Delta }}G={\rm{\Delta }}H-T{\rm{\Delta }}S$$

and since *ΔG* = 0 at the equilibrium temperature,

2 $${\rm{\Delta }}H=T{\rm{\Delta }}S$$

In pyrochlore oxides, the disordering proceeds gradually as a function of temperature via the formation of cation antisite (CA) and anion Frenkel pair (AFB) defects. For the general formula, [(A_1−x_B_x_)_2_]_*16d*_ [(B_1−x_A_x_)_2_]_*16c*_ (O_6−y_▯_y_)_*48f*_ (▯_1−y_O_y_)_*8a*_ O’_*8b*_ the cation/anion distribution is described by the following site interchange reactions.

For cation antisite disorder:

3 $$\begin{array}{llll}{A}_{16d}^{3+}+ & {B}_{16c}^{4+}\leftrightarrow  & {A}_{16c}^{3+}+ & {B}_{16d}^{4+}\\ ({1}\mbox{--}x) & ({1}\mbox{--}x) & x & x\end{array}$$

For anion Frenkel disorder:

4 $$\begin{array}{llll}{O}_{48f}^{2-}+ & {\square }_{8a}\leftrightarrow  & {O}_{8a}^{2-}+ & {\square }_{48f}\\ ({6}\mbox{--}y) & ({1}\mbox{--}y) & y & y\end{array}$$

Eq. (3) represents the cation exchange equilibrium reaction involving one mole of ‘A’ and one of ‘B.’ The equilibrium constant at a given temperature is written as:

5 $$\begin{array}{c}{K}_{cat}=\,\mathrm{ln}\,\begin{array}{c}[\frac{{x}^{2}}{{(1-x)}^{2}}];ln\,{K}_{cat}=2\,\mathrm{ln}\,\begin{array}{c}[\frac{x}{(1-x)}](for\,two\,moles\,of\,cations)\end{array}\end{array}\\ ln\,{K}_{cat}=4\,\mathrm{ln}\,\begin{array}{c}[\frac{x}{(1-x)}]\end{array}(for\,four\,moles\,of\,cations\,per\,{A}_{2}{B}_{2}{O}_{7}\,formula)\end{array}$$

Eq. (4) represents the anion exchange equilibrium reaction involving six moles of oxide ions and one mole of vacant anionic sites per A_2_B_2_O_7_ formula. The equilibrium constant at a given temperature is:

6 $$ln\,{K}_{anion}=\,\mathrm{ln}[\frac{{y}^{2}}{(6-y)(1-y)}]$$

The relation between equilibrium constant (*lnK*) and Gibbs free energy is,

7 $${\rm{\Delta }}G=-\,RT\,lnK$$

By substituting Eq. (6) & (7) in Eq. (1), the equilibrium constant can be written as follows

8 $$\mathrm{ln}\,K=-\,\frac{{\rm{\Delta }}G}{RT}=\frac{{\rm{\Delta }}S}{R}-\frac{{\rm{\Delta }}H}{RT}$$

The non-configurational entropy change associated with disordering is neglected, and we assumed the distribution of both cations and anions in a given sublattice is random. The configurational entropy (*S*_*conf*_) for a *PY* phase involving four moles of cations (2 moles of A-site + 2 moles of B-site), six moles of oxygens (6 moles of *48f*) and 1 mole of *8a* vacant sites (whereas the 1 mole of *8b* oxygens does not participate in Frenkel formation), is

9 $${S}_{conf,cat.}=-\,4R[(1-x)\mathrm{ln}(1-x)+x\,\mathrm{ln}\,x]$$

10 $${S}_{conf,ani.}=-\,R\{6[\frac{y}{6}ln\frac{y}{6}+(\frac{6-y}{6})\mathrm{ln}(\frac{6-y}{6})]+(1-y)\mathrm{ln}(1-y)+y\,\mathrm{ln}\,y\}$$

where ‘*x*’ is the fraction of *16d*-site cations in the *16c*-site and ‘*y*’ is the fraction of *48f* oxygens in *8a* vacant site. The total configuration entropy for both cation and anion distribution in a disordered *PY* (*DPY*) is given by *S*_*conf, DPY*_ = *S*_*conf, cat*_ + *S*_*Conf, ani*_. The configurational entropy of the fully disordered *DF* (i.e. A_1−*x*_B_*x*_O_2−*x*/2_▯_*x*/2_, *x* = 0.5 is fully disordered state) equivalent to *PY* stoichiometry including the disordering of *8b* oxygens is constructed as follows:

11 $${S}_{conf,DF}=-4R[(1-x)\mathrm{ln}(1-x)+xlnx+\frac{x}{2}ln\frac{x}{4}+(2-\frac{x}{2})\mathrm{ln}(1-\frac{x}{4})]$$

The configurational entropy for an ideal fully ordered *PY* phase is zero, and that of the completely disordered *DF* phase is 48.11 JK^−1^mol^−1^. The detailed configurational entropy calculations are provided in the supporting information.

The *S*_*conf*_ for Sm_2_Zr_2_O_7_, Eu_2_Zr_2_O_7_, and Gd_2_Zr_2_O_7_ based on site distribution at each temperature increment is given in the supporting information, Table S5. The entropy change for the phase transition from *PY* to *DF* is also calculated from the enthalpies obtained by DTA using Eq. (2). The calculated entropy changes during heating/cooling from DTA are 4.8 ± 0.9/−6.2 ± 0.9 and 3.5 ± 0.2/−4.0 ± 0.5 Jmol^−1^K^−1^ for Sm_2_Zr_2_O_7_ and Eu_2_Zr_2_O_7_, respectively (see, Table 3). Table 3 shows that the entropy change calculated from the enthalpies obtained by DTA using Eq. (2) is indeed similar to the *ΔS*_*conf*_ from site distribution. For example, for Eu_2_Zr_2_O_7_, the entropy change for ordering during cooling is −4.0 ± 0.5 from DTA and −6.1 ± 0.4 Jmol^−1^K^−1^ from the site distribution. Previously we reported the *PY*–*DF* phase transformation enthalpy for Eu_2_Zr_2_O_7_ to be 37.8 ± 3.1 kJ·mol^−1^ by measuring the enthalpy of the solution in a molten oxide solvent of *PY* and a laser-melt-quenched DF phase of the same composition. However, the structural state of the samples was not characterized in detail, so the results cannot be compared directly to those from the *in situ* high temperature DTA and site distributions in the present work.

Shamblin *et al*.^14,15^ and others^18,38^ suggest substantial short-range order at the nanoscale, described as a weberite-type structure that is derivative of fluorite structure bearing a higher degree of order than *DF*, in radiation-damaged *PY*. However, at present, there is no evidence, pro or con, of weberite-like ordering *in situ* at high temperatures, in either the partially disordered *PY* or *DF* phases. *In situ*, high-temperature neutron studies would be desirable to investigate this further, as X-ray diffraction gives little information about the short-range order on the oxygen sublattice. Without further information about range order, we proceed below to apply a thermodynamic model that assumes random distributions of the ions on each sublattice constrained by the measured site occupancies.

In the gradual disordering of *PY* with temperature, the favorable configurational entropy and the unfavorable energy (enthalpy) of disordering balance each other, resulting in greater disorder with increasing temperature. This balance can be described by an equilibrium constant analogous to that for spinel disordering^29^ to calculate the disordering enthalpy from the site distribution. Using this approach, the cation and anion disorder follow simple interchange reactions.

The following assumptions are made based on Navrotsky and Kleppa’s work on spinel disordering^32^:

12 $${\rm{\Delta }}H=x\,{\rm{\Delta }}{H}_{int};$$

13 $${\rm{\Delta }}H=y\,{\rm{\Delta }}{H}_{int};$$

(‘*x*’ and ‘*y*’ are fractions of cation and anion interchange)

14 $${\rm{\Delta }}S={S}_{confcat.}$$

*or*

15 $${S}_{confani.}$$

These equations imply that (a) the exchange of both cations and anions on each sublattice is ideal, *i.e*. there is negligible short-range order on each sublattice and no correlation between cation and anion distributions, (b) the enthalpy depends only on the interchange parameter and not on the extent of exchange, and (c) the entropy of disordering is configurational only. Substituting Eq. (11), (13) in (8) for cation antisite disorder and Eq. (12), (14) in (8) for anion Frenkel disorder and minimizing the free energy at a given temperature leads to the following relation for cation and anion interchange or disordering enthalpy:

16 $$For\,cation\,interchange,\,{\rm{\Delta }}{H}_{int,cat}=-\,4RT\,ln\frac{x}{(1-x)}$$

17 $$For\,anion\,interchange\,{\rm{\Delta }}{H}_{int,ani}=-\,RT\,ln\frac{{y}^{2}}{(6-y)(1-y)}$$

The molar enthalpies of interchange as a function of temperature using equations (15) and (16) are calculated for both CA and AFP disorder. Table 4 provides the calculated interchange enthalpy (enthalpy of disordering) values for RE_2_Zr_2_O_7_ [RE = Sm, Eu, and Gd]; the disordering appears to reach equilibrium (similar site occupancies on heating and cooling and consistent calculated interchange enthalpies over the range of temperatures marked in bold and italic).The calculations of equilibrium constant also left out the highest temperature points in the region of the transition from *PY* to *DF* since that transition appears to have a first order component and represents changes distinct from the equilibrium disordering within the *PY* phase. The average interchange enthalpies for cation disorder (CA) are 114.9 ± 9.6, 109.1 ± 13.6 and 90.9 ± 5.4 kJ·mol^−1^, and for anion Frenkel pair (AFP) disorder are 29.9 ± 3.7, 35.6 ± 3.5 and 35.9 ± 2.8 kJ.mol^−1^ for Sm, Eu, and Gd zirconates, respectively. The AFP interchange enthalpies show similar values for all three compositions and apparently do not depend on the size of the cation. The CA interchange enthalpy decrease with decreasing ‘A’ cation radius suggests greater disorder for smaller A-site cations at a given temperature. At any given temperature greater anion disorder exists than cation disorder.

**Table 4:** The calculated Interchange enthalpies based on site distribution for Sm_2_Zr_2_O_7_, Eu_2_Zr_2_O_7_ and Gd_2_Zr_2_O_7_ as a function of temperature.

Tv, K	Interchange Enthalpy (ΔH_int_), kJ.mol^−1^ Sm_2_Zr_2_O_7_		T, K	Interchange Enthalpy (ΔH_int_), kJ.mol^−1^ Eu_2_Zr_2_O_7_		Interchange Enthalpy (ΔH_int_), kJ.mol^−1^ Gd_2_Zr_2_O_7_	
	Cation	Anion		Cation	Anion	Cation	Anion
2423	80.3 ± 8.9		2073	78.1 ± 3.1	12.3 ± 5.0		
2323	94.9 ± 7.4	−18.3 ± 22.7	1973	***119.7*** ± ***6.6***	***37.9*** ± ***3.9***		
2223	93.3 ± 5.2	−19.3 ± 8.4	1873	***123.5*** ± ***3.5***	***38.1*** ± ***3.5***	40.5 ± 5.3	−27.9 ± 16.6
2123	98.3 ± 5.9	5.5 ± 5.6	1773	***119.6*** ± ***3.4***	***37.9*** ± ***3.1***	49.7 ± 3.4	−11.1 ± 23.1
2023	***120.0*** ± ***5.3***	***25.2*** ± ***4.9***	1673	***121.8*** ± ***3.7***	***38.9*** ± ***3.3***	***92.9*** ± ***4.0***	26.9 ± 6.1
1923	***128.3*** ± ***5.1***	***30.5*** ± ***4.6***	1573	***112.5*** ± ***3.4***	***38.5*** ± ***3.6***	***96.4*** ± ***3.2***	***39.3*** ± ***6.4***
1823	***120.2*** ± ***4.1***	***30.45*** ± ***3.5***	1473	***106.6*** ± ***3.2***	***34.9*** ± ***3.1***	***95.5*** ± ***4.3***	***36.8*** ± ***6.0***
1723	***115.9*** ± ***9.3***	***35.1*** ± ***3.6***	1373	***99.9*** ± ***3.0***	***29.2*** ± ***2.9***	***91.0*** ± ***3.1***	***38.1*** ± ***4.0***
1623	***115.5*** ± ***5.5***	***26.1*** ± ***3.9***	1273	***88.9*** ± ***2.6***	***32.0*** ± ***2.6***	***87.9*** ± ***3.1***	***35.3*** ± ***3.7***
1523	***103.4*** ± ***5.4***	***32.1*** ± ***5.8***	1173	***89.6*** ± ***2.8***	***33.2*** ± ***3.3***	***81.9*** ± ***2.9***	***34.3*** ± ***3.6***
1423	***101.2*** ± ***4.8***	21.2 ± 3.4	1073	80.6 ± 2.5	23.8 ± 2.3	74.1 ± 2.6	***31.4*** ± ***3.3***
1323	91.4 ± 4.3	18.1 ± 3.2	973	70.0 ± 2.1	25.3 ± 2.2	71.3 ± 2.6	25.6 ± 2.7
1223	75.0 ± 3.7	15.2 ± 3.0	873	54.1 ± 1.5	37.1 ± 3.7	63.2 ± 1.9	22.9 ± 2.5
1123	70.4 ± 3.1	18.1 ± 4.1	298	25.8 ± 1.3	15.7 ± 1.6	25.7 ± 1.3	9.7 ± 1.1
1023	67.9 ± 3.9	19.0 ± 5.0	—	—	—	—	—
298	15.9 ± 0.4	5.50 ± 0.7	—	—	—	—	—
**Avg. (** ***ΔH***_***int***_ **)**	**114.9** ± **9.6**	**29.9** ± **3.7**		**109.1** ± **13.6**	**35.6** ± **3.5**	**90.9** ± **5.4**	**35.9** ± **2.8**

Sm_2_Zr_2_O_7_ (heating) whereas Eu_2_Zr_2_O_7_ and Gd_2_Zr_2_O_7_ (cooling), the bold & italic face numbers show the state of equilibrium. The temperature of diffracted volume, T_v_ was calculated based on the observed differences in melting temperature obtained from cooling traces and *in situ* diffraction.

Since numerous computational studies report somewhat different defect energies in various *PY* oxides by applying force field (FF) and density functional theory (DFT) calculations, we will not compare our experimental values individually, but discuss general trends. The energies obtained by FFA calculations for RE = Sm, Eu and Gd range between 360 and 400 kJ·mol^−1^ for cation disorder and 480 and 560 kJ.mol^−1^ for anion Frenkel disorder^20,21^. The FF modeling studies consider a single defect formation energies at 0 K, whereas in the current study the defect energy values are obtained on a structure that already contains a significant amount of disorder at room temperature (5–10%) and even more in the range of the measurements. So, the direct quantitative comparison may not be appropriate. The recent DFT calculations show lower values ranging between 160 and 200 kJ·mol^−1^ for cation disorder and negative values for anion Frenkel disorder, especially for compositions that lie near the *PY*–*DF* phase boundary^25^.

The current experimentally derived values range between 90 and 140 kJ·mol^−1^ for CA, and 30–36 kJ·mol^−1^ for AFP disorder, reasonably similar to the DFT calculations^25^. Both computational and experimental trends follow radius ratio r_A_/r_Zr_ rules; i.e. the phase transition to *DF* shifts to lower temperatures as the radius of the A-site cation decreases and the lowest interchange enthalpy is associated with Gd_2_Zr_2_O_7_, which shows the greatest tendency to disorder.

## Conclusions

We investigated the temperature-induced order–disorder phase transition in zirconate pyrochlores using a combination of *in situ* synchrotron X-ray diffraction and aerodynamic levitation with laser heating up to the melting points. The obtained diffraction pattern at each temperature was subjected to Rietveld analysis to extract the change in lattice constant, antisite cation/anion occupancies and phase transition temperatures. The lattice constant changed smoothly as a function of temperature, and the calculated thermal expansion coefficients fall in the range of 8.6 to 10.8 × 10^−6^ K^−1^. For the first time, we have experimentally determined the cation and anion disorder enthalpies based on a simple thermodynamic model and antisite cation and anion Frenkel occupancies. The anion Frenkel disorder enthalpies are lower than those for cation antisite disorder. This thermodynamic model can easily be extended to a variety of *PY* compounds to understand the disordering energetics, thereby tuning their functional properties. Diffraction on laser-heated aerodynamically levitated samples allows precise structural characterization and enables determination of disordering enthalpies and thermal expansion close to the melting point.

## Methods

### Sample preparation and characterization

A series of stoichiometric rare earth pyrochlores (*PY*) with the general formula A_2_B_2_O_7_, where A = Rare earth (RE), B = Zr, Hf was synthesized by laser hearth melting. The starting materials used were high purity ZrO_2_ (Aldrich; 99.99%), HfO_2_ (Alfa Aesar; 99.9%), and RE_2_O_3_ (Alfa Aesar, 99.9% or higher). Prior to synthesis, the RE_2_O_3_ oxides were heated overnight at 1000 °C to remove any adsorbed moisture and carbon dioxide. Desired amounts of RE_2_O_3_ [RE: La, Nd, Sm, Eu, Gd] and ZrO_2_ or HfO_2_ were finely ground and melted in a copper hearth into oblate spheroids 1.5–2.5 mm in diameter with a CO_2_ laser using an experimental setup described in detail elsewhere^39,40^. The cooling profiles on crystallization were recorded using a spectropyrometer on samples aerodynamically levitated in air flow and analysed for thermal arrests. The composition and homogeneity of the samples were determined by wavelength dispersive electron probe microanalysis (Cameca SX100, Gennevilliers, France). The instrument was operated at an accelerating voltage of 15 kV, with a 20 nA beam current with a spot size of 1 µm. The calculated compositions are an average of several data points per sample.

### Thermal Analysis

Ultra-high-temperature differential thermal analysis (DTA) was carried out on Sm_2_Zr_2_O_7_, Eu_2_Zr_2_O_7_ and Gd_2_Zr_2_O_7_ using a Setaram Setsys 2400 (Caluire, France). Tungsten crucibles with lids were used in an argon flow of 20 mL/min. The laser melted samples were sealed in tungsten crucible using a custom semiautomatic welding chamber (Miller, Appleton, WI) to prevent carbon contamination. To account for thermocouple aging effects, temperature and sensitivity calibrations were performed before and after the sample measurements by melting Al_2_O_3_ using a procedure described elsewhere^41^.

### High-temperature *in situ* diffraction on levitated samples

To gain insight into the essential structural features associated with temperature change, and to capture the gradual disordering in complex *PY* oxides, all the compositions were subjected to incremental heating using aerodynamic levitation and laser heating followed by *in situ* synchrotron diffraction at each temperature. The high-temperature *in situ* synchrotron X-ray diffraction experiments were performed at the Advanced Photon Source, Argonne National Laboratory, Illinois (USA) at beamlines 11-ID-C and 6-ID-D^42^. Two different wavelengths were used for the high-energy X-ray measurements, 0.139397 Å in beamline 6-ID-D, and 0.10798 Å in beamline 11-ID-C. A Perkin Elmer XRD 1621 high-sensitivity fast-readout large-area flat panel detector based on amorphous silicon was used to collect the scattered high-energy X-rays^43^. The sample–detector distance and tilt angle were set to the maximum distance (~1 m) to increase resolution. The sample temperature was measured by a single-band pyrometer (IR-CAS3CS; Chino, Tokyo, Japan) with emissivity set to 0.93. An unconditioned beam from a Synrad CO_2_ laser was used for sample heating. The experimental arrangement, including the aerodynamic levitator, was described in detail by Weber *et al*.^44–47^. The diffraction experiments at 11-ID-C were performed on samples 1.01.2 mm in diameter followed by experiments at 6-ID-D on 2.42.6 mm samples. The difference in bead dimension, necessitating slightly different acquisition and processing conditions, was the result of a lower power 100 W CO_2_ laser used for the earlier experiments. The 2D X-ray images were collected by summing up 30 frames with 1.0 s exposures at beamline 11-ID-C, and 100 frames with 0.1 s exposures at beamline 6-ID-D. The obtained synchrotron diffraction rings were integrated using Fit2D^48,49^ processing software. The sample-to-detector distance, beam position, and detector tilt was refined using the CeO_2_ standard. In the case of the smaller diameter samples, the diffraction rings were masked for the nozzle background. The temperature of the diffracted volume was calculated from the surface temperature measured by a pyrometer, with correction determined from measured melting temperatures and the appearance of the amorphous halo in the X-ray diffraction pattern, as described previously^40,50^.

### Rietveld structure refinement

The high-temperature *in situ* synchrotron diffraction data was grouped into two sets based on the quality of data obtained and beamline used. A complete Rietveld structure refinement was performed for high-temperature diffraction data on Sm_2_Zr_2_O_7_, Eu_2_Zr_2_O_7_, and Gd_2_Zr_2_O_7_ using the FULLPROF program^51^. All the refinements were performed in the following manner. The background was selected manually, and a pseudo-Voigt function was used to model the peak shape. After successful refinement of the scale factor, lattice constant, peak shape, the antisite cation distribution (between *16d/16c* crystallographic sites) and anion Frenkel (vacancy on a 48*f* site and oxygen on 8*a* site^21^) was refined under the constraint of stoichiometric composition. Finally, the *48f* ‘*x*’ positional and isothermal parameters were refined. For the high-temperature diffraction data from the La_2_Hf_2_O_7_, Nd_2_Zr_2_O_7_, Nd_2_Hf_2_O_7_ and Sm_2_Hf_2_O_7_ phases_,_ pattern matching was performed using the Le Bail method^52^ for unit cell refinement.

## Electronic supplementary material

Supplementary Information
