# Supplementary material for: Probing disorder in pyrochlore oxides using in situ synchrotron diffraction from levitated solids–A thermodynamic perspective
Source: Sci Rep. 2018 Jul 13;8:10658. doi: 10.1038/s41598-018-28877-x (PMC6045670; doi:10.1038/s41598-018-28877-x)
Supplement: Supplementary file 1 — Supplementary Information [file 41598_2018_28877_MOESM1_ESM.docx]

**Supplementary Information**

**Probing disorder in pyrochlore oxides using *in situ* synchrotron diffraction from levitated solids – A thermodynamic perspective**

**Pardha S Maram^‡^, Sergey V. Ushakov^‡^, Richard J.K. Weber^†, §^, Chris J. Benmore^†^, and Alexandra Navrotsky*^‡^**

**Configurational Entropy calculations:**

For a random distribution of ‘*n’* mole of ions over ‘*n’* mole of sites, the configurational entropy will be calculated using the Boltzmann statistical distribution relation

S_conf_ = -*n*R(*z*_A_ ln*z*_A_ + *z*_B_ ln*z*_B_)

where ‘*n*’ is the number of sites on which random distribution occurs. *z*_A_ and *z*_B_ are mole fractions of A and B.

Ideally, the general formula of oxide pyrochlore is written as A_2_B_2_O_6_O'. The space group is *Fd3m*, with the unit cell containing eight molecules (Z = 8) and four nonequivalent sites. The atoms are occupying the following special positions: A at *16d*, B at *16c*, O at *48f*, O' at *8b* and a vacancy at *8a*. The cation antisite disorder proceeds by occupying ‘x’ moles of B-site in A-site and vis-à-vis. During the anion sublattice disorder (forming oxygen Frenkel), only the six oxygens at *48f* involve in disordering process whereas the O' at *8b* does not participate in the disordering [Ewing et al. (2004), Yan Li et al. (2015)]. The oxygen Frenkel defect consists of a vacancy on a *48f* site and interstitial oxygen on a vacant *8a* site, *i.e.* (O_6-yy_)*_48f_* (_1-y_O_y_)*_8a_* O'*_8b_*. While writing configurational entropy equation for Frenkel defect formation, we consider only six oxygens and one vacant site. The calculation of configurational entropy for a partial disorder pyrochlore is calculated as follows.

R.C. Ewing, W.J. Weber, J. Lian, “Nuclear waste disposal-pyrochlore (A_2_B_2_O_7_): Nuclear waste form for the immobilization of plutonium and minor actinides. *J. Appl. Phys.* 95 (2004) 5949

Yan Li, Piotr M. Kowalski, George Beridze, April R. Birnie, Sarah Finkeldei, Dirk Bosbach, “Defect formation energies in A_2_B_2_O_7_ pyrochlores”, *Scr. Mater*. 107 (2015) 18

**Configurational Entropy of disordered pyrochlore (*DPY*):**

For the general formula, [(A_1-x_B_x_)_2_]*_16d_* [(B_1-x_A_x_)_2_]*_16c_* (O_6-yy_)*_48f_* (_1-y_O_y_)*_8a_* O'*_8b_* the cation/anion configurational entropy is computed as follows:

**Cation sublattice, [(A_1-x_B_x_)_2_]*_16d_* [(B_1-x_A_x_)_2_]*_16c_***

**Step 1 (16d)**

‘*x*’ mole of B occupied in A- site (*16d*), the configurational entropy for this 2 mole of *16d* A-sites can be written as

S*_16d site_* = $-R\left[ (1-x)\ln(1-x)+x\ln x \right]$ (for one mole of A-sites)

S*_16d site_* = $-2R\left[ (1-x)\ln(1-x)+x\ln x \right]$ (for two mole of A-sites)

**Step 2 (16c)**

‘*x*’ mole of A occupied in B- site (*16c*), the configurational entropy for this 2 mole of *16c* sites can be written as

S*_16C site_* = $-2R\left[ (1-x)\ln(1-x)+x\ln x \right]$

The total cation configurational entropy, *S_conf, cat_* = S_16d site_ + S_16C site_

*S_conf, cat_* = $-4R\left[ (1-x\ln(1-x)+x\ln x \right]$

**Anion sublattice**

In an ideal pyrochlore 6 mole *48f* sites and 1 mole vacant 8a sites participate in anion Frenkel formation i.e. [(A_1-x_B_x_)]_2_*_16d_* [(A_1-x_B_x_)_2_]*_16c_* (O_6-yy_)*_48f_* (_1-y_O_y_)*_8a_* O'*_8b_*

**Step 1 (*48f*)**

‘*y*’ mole of *8a* vacancy occupied in *48f*- site, the configurational entropy for this 6 mole of *48f* sites can be written as

S*_48f site_* = $-6R\left[ \frac{(6-y)}{6}\ln\frac{(6-y)}{6}+\frac{y}{6}\ln\frac{y}{6} \right]$

**Step 2 (*8a*)**

‘*y*’ mole of *48f oxygen* occupied in *8a*- site, the configurational entropy for this 1 mole of *8a* sites can be written as

S*_8a site_* = $-R\left[ (1-y)\ln(1-y)+y\ln y \right]$

The total anion Frenkel configurational entropy, *S_conf, ani_* = S*_48f_* _site_ + S*_8a_* _site_

$$S_{conf, ani.}=-R\left\{ 6\left[ \frac{y}{6}ln\frac{y}{6}+\left( \frac{6-y}{6} \right)\ln\left( \frac{6-y}{6} \right) \right]+\left( 1-y \right)\ln\left( 1-y \right)+y\ln y \right\}$$

Configurational Entropy of disordered pyrochlore, $S_{conf, DPY}$ = $S_{conf, cat}+S_{conf, ani.}$

**Configurational Entropy of defect fluorite (DF):**

The general formula of completely disordered defect fluorite, A_1-x_B_x_O_2-x/2x/2_ and the doping creates vacancies on anion sublattice.

The configurational entropy for the completely disordered DF is computed as follows:

$S_{conf, DF}=-R[\left( 1-x \right)\ln\left( 1-x \right)+xlnx+ \frac{x}{2}ln\frac{x}{4}+\left( 2-\frac{x}{2} \right)\ln\left( 1-\frac{x}{4} \right)]$ (For one mole of *DF*)

$S_{conf, DF}=-4R[\left( 1-x \right)\ln\left( 1-x \right)+xlnx+ \frac{x}{2}ln\frac{x}{4}+\left( 2-\frac{x}{2} \right)\ln\left( 1-\frac{x}{4} \right)]$ (Equivalent to PY formula)

The configurational entropy for fully disordered DF i.e. at *x* = 0.5, $S_{conf, DF}=48.11 kJ/mol$





**Figure S1:** Room temperature synchrotron *X-ray* diffraction pattern of all the melt-quenched compositions, the synchrotron wavelength for (a to e) is 0.139397 Å (beamline, 6-ID-D) whereas synchrotron wavelength for (f to g) is 0.10798 Å (beamline, 11-ID-C)





**Figure S2:** *In situ* synchrotron diffraction pattern of La_2_Hf_2_O_7_ as a function of temperature, the superlattice reflections are present till melting point at 2673K. The temperature of diffracted volume, Tv was calculated based on the observed difference in melting temperature from cooling trace and *in situ* diffraction.





**Figure S3:** *In situ* synchrotron diffraction pattern of Nd_2_Hf_2_O_7_ as a function of temperature, the superlattice reflections are present till melting at 2773K. The temperature of diffracted volume, Tv was calculated based on the observed difference in melting temperature from cooling trace and *in situ* diffraction.





**Figure S4:** *In situ* synchrotron diffraction pattern of Nd_2_Zr_2_O_7_ as a function of temperature, the (311) superlattice reflection present till 2523K but the remaining (111), (331) and (511) present till melting point at 2673K. The temperature of diffracted volume, Tv was calculated based on the observed difference in melting temperature from cooling trace and *in situ* diffraction.





**Figure S5:** *In situ* synchrotron diffraction pattern of Sm_2_Hf_2_O_7_ as a function of temperature. The superlattice *PY* reflections intensified up to 2123 K, but further increase in temperature weakened the superlattice reflections, which completely disappeared, indicating transformation to *DF* just before melting at 2823 K. The temperature of diffracted volume, Tv was calculated based on the observed difference in melting temperature from cooling trace and *in situ* diffraction.





**Figure S6:** *In situ* synchrotron diffraction pattern of Sm_2_Zr_2_O_7_ as a function of temperature, the superlattice reflections disappeared indicating *PY* – *DF* phase transformation at 2323K and melting occurred at 2823K. The temperature of diffracted volume, Tv was calculated based on the observed difference in melting temperature from cooling trace and *in situ* diffraction.





**Figure S7:** *In situ* synchrotron diffraction pattern of Eu_2_Zr_2_O_7_ during heating and cooling, the starting phase of the diffraction experiment is *DF*. During heating the superlattice reflections started to appear around 1573K intensified further increase in temperature and disappeared at 2173K while cooling from 2373K, *PY* reflections started to appear at 2073K. The Eu_2_Zr_2_O_7_ exhibited reversible *PY* – *DF* phase transition at 2173K during heating and 2073K during cooling.





**Figure S8:** *In situ* synchrotron diffraction pattern of Gd_2_Zr_2_O_7_ during heating and cooling, the starting phase of the diffraction experiment is *DF*. During heating the *PY* reflections started to appear around 1473K intensified further increase in temperature and disappeared at 1873K while cooling from 2573K, *PY* reflections started to appear at 1773K.

**Table S1. Results of Le Bail lattice constant refinement for *Py* compositions**

| **Tv, K** | **La_2_Hf_2_O_7_** | | **Nd_2_Hf_2_O_7_** | | **Nd_2_Zr_2_O_7_** | | **Sm_2_Hf_2_O_7_** | |
| --- | --- | --- | --- | --- | --- | --- | --- | --- |
|  | **a, Å** | **χ2** | **a, Å** | **χ2** | **a, Å** | **χ2** | **a, Å** | **χ2** |
| 298 | 10.7916(1) | 7.2 | 10.6455(1) | 3.7 | 10.6606(1) | 5.2 | 10.5838(1) | 7.9 |
| 1023 | 10.8808(1) | 12.7 | 10.7434(1) | 6.1 | 10.7672(1) | 7.6 | 10.6823(1) | 8.7 |
| 1123 | 10.8888(1) | 11.3 | 10.7525(1) | 6.2 | 10.7741(1) | 7.2 | 10.6926(2) | 13.7 |
| 1223 | 10.8975(1) | 7.7 | 10.7623(1) | 5.9 | 10.7814(1) | 8.7 | 10.6998(2) | 11.9 |
| 1323 | 10.9068(1) | 8.0 | 10.7727(1) | 5.8 | 10.7934(1) | 5.3 | 10.7038(1) | 6.2 |
| 1423 | 10.9157(1) | 7.3 | 10.7818(1) | 6.1 | 10.8009(1) | 5.1 | 10.7175(1) | 9.1 |
| 1523 | 10.9254(1) | 6.6 | 10.7914(1) | 7.4 | 10.8117(1) | 5.3 | 10.7267(2) | 12.4 |
| 1623 | 10.9328(1) | 6.3 | 10.8009(1) | 6.8 | 10.8269(1) | 6.7 | 10.7449(1) | 4.4 |
| 1723 | 10.9407(1) | 4.7 | 10.8106(1) | 6.7 | 10.8340(1) | 5.1 | 10.7534(1) | 4.6 |
| 1823 | 10.9472(1) | 5.0 | 10.8194(1) | 5.7 | 10.8457(1) | 4.3 | 10.7619(1) | 6.4 |
| 1923 | 10.9537(1) | 6.4 | 10.8317(1) | 5.1 | 10.8571(1) | 3.6 | 10.7723(1) | 5.8 |
| 2023 | 10.9622(1) | 7.8 | 10.8386(1) | 5.9 | 10.8693(1) | 3.3 | 10.7805(1) | 5.3 |
| 2123 | 10.9745(1) | 6.0 | 10.8496(1) | 5.3 | 10.8816(1) | 2.8 | 10.7883(1) | 5.8 |
| 2223 | 10.9867(1) | 4.4 | 10.8578(1) | 4.2 | 10.8895(1) | 3.2 | 10.7958(2) | 9.2 |
| 2323 | 10.9870(1) | 6.7 | 10.8703(1) | 2.6 | 10.8958(1) | 3.2 | 10.8029(1) | 5.5 |
| 2423 | 10.9925(1) | 6.4 | 10.8788(1) | 3.1 | 10.8983(1) | 1.3 | 10.8119(1) | 4.9 |
| 2523 | 11.0018(2) | 7.5 | 10.8862(2) | 7.4 | 10.8963(1) | 2.6 | 10.8185(4) | 12.7 |
| 2623 | 11.0089(2) | 7.2 | 10.8871(1) | 3.6 | 10.9116(1)† | 0.6 | 10.8234(2) | 9.9 |
| 2723 | − | − | 10.8886(1) | 2.4 | − | − | 10.8257(2) | 3.1 |
| 2823 | − | − | − | − | − | − | 10.8246(8) | 1.9 |

†The lattice constant for defect fluorite was doubled for direct comparison

| **Tv, K** | **†Lattice constant, Å** | **Refined Occupancies for pyrochlore phase** | | | | **^‡^*48f ‘x’*** | **% Cationic disorder** | **%**  **Anion disorder** | **Bragg R** | **R_f_-factor** | **χ2** |
| --- | --- | --- | --- | --- | --- | --- | --- | --- | --- | --- | --- |
|  |  | **Sm_16d_** | **Zr_16d_** | **O_48f_** | **O_8a_** |  |  |  |  |  |  |
| 298 | 10.5965(4) | - | - | - | - | 0.346(1) | - | - | 4.6 | 3.2 | 5.8 |
| 1023 | 10.7044(4) | 1.76(2) | 0.24(2) | 5.47(10) | 0.53(10) | 0.355(3) | 12.0(1.2) | 8.8(1.6) | 1.7 | 1.1 | 6.2 |
| 1123 | 10.7169(2) | 1.74(2) | 0.26(2) | 5.42(7) | 0.58(7) | 0.350(2) | 13.2(1.0) | 9.6(1.2) | 3.6 | 5.2 | 4.4 |
| 1223 | 10.7330(3) | 1.73(2) | 0.27(2) | 5.35(5) | 0.65(5) | 0.350(2) | 13.7(1.1) | 10.8(0.8) | 6.4 | 6.6 | 6.0 |
| 1323 | 10.7343(2) | 1.78(2) | 0.22(2) | 5.38(5) | 0.62(5) | 0.347(2) | 11.2(1.0) | 10.4(0.8) | 3.2 | 5.7 | 4.8 |
| 1423 | 10.7506(3) | 1.79(2) | 0.21(2) | 5.40(5) | 0.60(5) | 0.347(2) | 10.6(1.0) | 10.0(0.8) | 5.1 | 6.4 | 5.4 |
| 1523 | 10.7639(2) | 1.77(2) | 0.23(2) | 5.52(7) | 0.48(7) | 0.348(1) | 11.5(1.1) | 8.0(1.2) | 3.9 | 6.9 | 4.7 |
| 1623 | 10.7743(2) | 1.79(2) | 0.21(2) | 5.42(5) | 0.58(5) | 0.346(2) | 10.6(1.0) | 9.6(0.8) | 5.4 | 6.7 | 6.1 |
| 1723 | 10.7833(2) | 1.76(2) | 0.24(2) | 5.51(4) | 0.49(4) | 0.348(1) | 11.8(1.7) | 8.2(0.7) | 5.0 | 6.6 | 4.5 |
| 1823 | 10.7933(2) | 1.76(1) | 0.24(1) | 5.44(4) | 0.56(4) | 0.348(1) | 12.1(0.7) | 9.4(0.6) | 4.7 | 6.9 | 3.5 |
| 1923 | 10.8023(3) | 1.76(2) | 0.24(2) | 5.42(5) | 0.58(5) | 0.349(2) | 11.9(0.8) | 9.7(0.8) | 8.0 | 7.4 | 5.8 |
| 2023 | 10.8117(3) | 1.71(2) | 0.29(2) | 5.35(5) | 0.65(5) | 0.345(2) | 14.4(1.0) | 10.8(0.8) | 7.8 | 8.4 | 5.1 |
| 2123 | 10.8216(3) | 1.60(3) | 0.40(3) | 5.18(7) | 0.82(7) | 0.352(2) | 19.9(1.3) | 13.6(1.2) | 9.2 | 8.7 | 7.3 |
| 2223 | 10.8288(2) | 1.56(2) | 0.44(2) | 5.06(2) | 0.94(2) | 0.354(2) | 22.1(1.2) | 15.6(0.4) | 3.9 | 7.4 | 2.8 |
| 2323 | 10.8354(5) | 1.55(3) | 0.45(4) | 5.09(10) | 0.91(10) | 0.356(3) | 22.7(1.7) | 15.2(1.6) | 10.0 | 6.4 | 6.7 |
| 2423 | 10.8431(5) | 1.46(4) | 0.54(4) | 5.00(10) | 1.00(10) | 0.361(5) | 27.0(2.2) | 16.8(1.6) | 9.7 | 5.7 | 7.6 |
| 2523 | 10.8451(5)† | − | − | − | − | − | − | − | 10.0 | 7.3 | 6.7 |
| 2623 | 10.8577(4)† | − | − | − | − | − | − | − | 5.5 | 11.6 | 11.2 |
| 2723 | 10.8759(2)† | − | − | − | − | − | − | − | 6.3 | 6.6 | 3.8 |

**Table S2. Results of Rietveld structure refinement on heating Sm_2_Zr_2_O_7_**

†The lattice constant for defect fluorite was doubled for direct comparison

**Table S3. Results of Rietveld structure refinement on heating Gd_2_Zr_2_O_7_**

| **T, K** | **†Lattice constant, Å** | **Refined Occupancies for pyrochlore phase** | | | | **^‡^*48f ‘x’*** | **% Cationic disorder** | **%**  **Anion disorder** | **Bragg R** | **R_f_-factor** | **χ2** |
| --- | --- | --- | --- | --- | --- | --- | --- | --- | --- | --- | --- |
|  |  | **Gd_16d_** | **Zr_16d_** | **O_48f_** | **O_8a_** |  |  |  |  |  |  |
| 298 | 10.5258(1) | - | - | - | - | - |  |  | 5.18 | 2.92 | 1.71 |
| 873 | 10.6174(2) | - | - | - | - | - |  |  | 2.79 | 1.61 | 1.03 |
| 973 | 10.6232(2) | - | - | - | - | - |  |  | 2.82 | 1.74 | 1.03 |
| 1073 | 10.6282(2) | - | - | - | - | - |  |  | 3.29 | 2.58 | 1.62 |
| 1173 | 10.6411(2) | - | - | - | - | - |  |  | 3.85 | 2.71 | 1.93 |
| 1273 | 10.6451(2) | - | - | - | - | - |  |  | 4.49 | 2.55 | 2.29 |
| 1373 | 10.6610(1) | - | - | - | - | - |  |  | 2.81 | 2.09 | 1.42 |
| 1473 | 10.6701(1) | 1.32(4) | 0.68(4) | 5.52(7) | 0.48(7) | 0.365(2) | 34.2(2.0) | 8.0(1.2) | 4.67 | 8.37 | 2.51 |
| 1573 | 10.6796(1) | 1.38(3) | 0.62(3) | 5.50(10) | 0.50(7) | 0.368(3) | 30.8(1.7) | 8.4(1.2) | 4.7 | 7.43 | 2.43 |
| 1673 | 10.6898(1) | 1.41(3) | 0.59(3) | 5.62(7) | 0.38(7) | 0.363(2) | 29.6(1.3) | 6.4(1.2) | 5.05 | 7.79 | 2.24 |
| 1773 | 10.6994(1) | 1.31(3) | 0.69(3) | 5.59(5) | 0.41(5) | 0.369(3) | 34.7(1.7) | 6.8(0.8) | 5.21 | 8.18 | 1.77 |
| 1873 | 10.7049(1) | 1.25(2) | 0.75(2) | 5.62(4) | 0.38(4) | 0.365(4) | 37.7(1.1) | 6.4(0.6) | 3.79 | 6.21 | 1.29 |
| 1973 | 10.7116(1) | 1.03(3) | 0.97(3) | 5.06(4) | 0.94(4) | 0.375(5) | 48.5(1.6) | 15.6(0.6) | 4.31 | 5.7 | 1.27 |
| 2073 | 10.7312(1) | - | - | - | - | - |  |  | 4.68 | 6.29 | 1.48 |
| 2173 | 10.7385(1) | - | - | - | - |  |  |  | 2.97 | 2.01 | 1.8 |
| 2273 | 10.7469(3) | - | - | - | - | - |  |  | 3.00 | 2.99 | 0.88 |
| 2373 | 10.7665(2) | - | - | - | - | - |  |  | 4.92 | 3.54 | 1.38 |
| 2473 | 10.7684(1) | - | - | - | - | - |  |  | 4.37 | 4.93 | 1.21 |
| 2573 | 10.7644(1) | - | - | - | - | - |  |  | 3.73 | 3.81 | 1.70 |

**†** The lattice constant for defect fluorite phase (298-1373K) and (2073-2523K) was doubled for direct comparison. **^‡^**48f oxygen ‘x’- positional parameter in pyrochlore structure, numbers in parenthesis are errors in the last digit obtained from least squares fitting of structure refinement.

**Table S4. Results of Rietveld structure refinement on cooling Gd_2_Zr_2_O_7_**

| **T, K** | **†Lattice constant, Å** | **Refined Occupancies for pyrochlore phase** | | | | **^‡^*48f ‘x’*** | **% Cationic disorder** | **%**  **Anion disorder** | **Bragg R** | **R_f_-factor** | **χ2** |
| --- | --- | --- | --- | --- | --- | --- | --- | --- | --- | --- | --- |
|  |  | **Gd_16d_** | **Zr_16d_** | **O_48f_** | **O_8a_** |  |  |  |  |  |  |
| 2373 | 10.7627(1) | - | - | - | - |  |  |  | 3.37 | 5.71 | 1.01 |
| 2273 | 10.7660(1) | - | - | - | - |  |  |  | 5.27 | 6.26 | 1.63 |
| 2173 | 10.7461(1) | - | - | - | - |  |  |  | 5.89 | 3.66 | 1.38 |
| 2073 | 10.7225(1) | - | - | - | - |  |  |  | 6.12 | 4.28 | 1.71 |
| 1973 | 10.7168(1) | - | - | - | - |  |  |  | 5.53 | 5.99 | 4.09 |
| 1873 | 10.7067(1) | 1.32(4) | 0.68(4) | 4.99(10) | 1.01(10) | 0.367(5) | 34.3(1.9) | 16.8(1.6) | 6.37 | 7.45 | 2.29 |
| 1773 | 10.7013(1) | 1.40(2) | 0.60(2) | 5.11(10) | 0.89(10) | 0.362(3) | 30.1(1.2) | 14.8(1.6) | 5.97 | 6.72 | 1.63 |
| 1673 | 10.6928(1) | 1.68(2) | 0.32(2) | 5.42(7) | 0.58(7) | 0.353(2) | 15.8(1.0) | 9.6(1.2) | 6.10 | 8.46 | 3.17 |
| 1573 | 10.6827(1) | 1.73(1) | 0.27(1) | 5.59(7) | 0.41(7) | 0.353(2) | 13.7(0.7) | 6.8(1.2) | 6.28 | 8.48 | 3.12 |
| 1473 | 10.6731(1) | 1.75(2) | 0.25(2) | 5.59(7) | 0.41(7) | 0.353(2) | 12.5(1.0) | 6.8(1.2) | 8.44 | 11.0 | 6.35 |
| 1373 | 10.6700(1) | 1.76(1) | 0.24(1) | 5.64(5) | 0.36(5) | 0.352(2) | 12.0(0.7) | 6.0(0.8) | 5.78 | 8.01 | 3.3 |
| 1273 | 10.6582(1) | 1.78(1) | 0.22(1) | 5.64(5) | 0.36(5) | 0.351(1) | 11.2(0.7 | 6.0(0.8) | 6.02 | 8.94 | 3.38 |
| 1173 | 10.6493(1) | 1.78(1) | 0.22(1) | 5.66(5) | 0.34(5) | 0.350(1) | 10.9(0.7) | 5.6(0.8) | 5.70 | 7.61 | 3.39 |
| 1073 | 10.6438(1) | 1.78(1) | 0.22(1) | 5.66(5) | 0.34(5) | 0.351(1) | 11.2(0.7) | 5.6(0.8) | 5.39 | 7.16 | 3.25 |
| 973 | 10.6315(1) | 1.80(1) | 0.20(1) | 5.62(5) | 0.38(5) | 0.350(1) | 10.0(0.7) | 6.4(0.8) | 5.06 | 6.81 | 3.69 |
| 873 | 10.6090(1) | 1.80(1) | 0.20(1) | 5.62(5) | 0.38(5) | 0.350(1) | 10.2(0.6) | 6.4(0.8) | 4.99 | 7.94 | 3.33 |
| 298 | 10.5296(1) | 1.86(2) | 0.14(2) | 5.71(5) | 0.29(5) | 0.345(2) | 7.0(0.8) | 4.8(0.8) | 8.80 | 9.13 | 5.87 |

**†** The lattice constant for defect fluorite (2373-1973K) was doubled for direct comparison. **^‡^**48f oxygen ‘x’- positional parameter in pyrochlore structure, numbers in parenthesis are errors in the last digit obtained from least squares fitting of structure refinement

**Table S5. The calculated configurational entropy based on site distribution obtained from Rietveld analysis**

| **Tv, K** | **Sm_2_Zr_2_O_7_ (J.K^-1^.mol^-1^)** | | | **T, K** | **Eu_2_Zr_2_O_7_ (J.K^-1^.mol^-1^)** | | | **Gd_2_Zr_2_O_7_ (J.K^-1^.mol^-1^)** | | |
| --- | --- | --- | --- | --- | --- | --- | --- | --- | --- | --- |
|  | **Cation** | **Anion** | **Total** |  | **Cation** | **Anion** | **Total** | **Cation** | **Anion** | **Total** |
| 2423 | 19.40±0.72 | 22.64±0.43 | 42.04±0.84 | 2073 | 18.46±0.32 | 23.59±0.25 | 42.05±0.40 |  |  |  |
| 2323 | 17.08±0.69 | 23.74±0.47 | 41.54±0.83 | 1973 | 13.42±0.73 | 20.34±0.74 | 33.76±1.04 |  |  |  |
| 2223 | 17.56±0.50 | 23.58±0.20 | 41.13±0.54 | 1873 | 12.28±0.40 | 19.91±0.73 | 32.19±0.83 | 21.39±0.41 | 21.89±0.54 | 42.73±0.60 |
| 2123 | 16.60±0.61 | 23.80±0.23 | 40.41±0.65 | 1773 | 11.96±0.40 | 19.51±0.72 | 31.47±0.82 | 20.35±0.34 | 19.85±1.06 | 42.24±0.64 |
| 2023 | 13.71±0.57 | 21.52±0.76 | 36.18±0.83 | 1673 | 10.87±0.44 | 18.71±0.84 | 29.58±0.95 | 14.53±0.53 | 16.71±1.66 | 34.38±1.18 |
| 1923 | 12.12±0.56 | 21.24±0.64 | 33.64±0.95 | 1573 | 11.13±0.43 | 18.19±1.00 | 29.32±1.09 | 13.27±0.44 | 16.71±1.66 | 29.98±1.71 |
| 1823 | 12.28±0.47 | 19.91±0.83 | 33.52±0.80 | 1473 | 10.96±0.43 | 18.54±0.91 | 29.49±1.01 | 12.52±0.62 | 15.54±1.23 | 29.22±1.77 |
| 1723 | 12.04±1.13 | 21.44±0.77 | 31.95±1.40 | 1373 | 10.87±0.44 | 19.56±0.87 | 30.43±0.97 | 12.20±0.48 | 15.54±1.23 | 27.75±1.32 |
| 1623 | 11.21±0.68 | 19.66±1.51 | 32.65±1.03 | 1273 | 11.47±0.42 | 17.89±0.91 | 29.36±1.00 | 11.63±0.50 | 14.91±1.30 | 27.18±1.33 |
| 1523 | 11.88±0.73 | 21.81±0.72 | 31.54±1.68 | 1173 | 10.18±0.46 | 16.49±1.29 | 26.66±1.37 | 11.47±0.50 | 14.91±1.30 | 26.38±1.39 |
| 1423 | 11.21±0.68 | 22.15±0.66 | 33.03±0.99 | 1073 | 10.43±0.45 | 19.20±0.90 | 29.62±1.01 | 11.63±0.50 | 16.14±1.16 | 26.55±1.39 |
| 1323 | 11.63±0.66 | 22.47±0.60 | 33.79±0.93 | 973 | 11.04±0.43 | 17.53±1.00 | 28.57±1.09 | 10.78±0.53 | 16.14±1.16 | 26.92±1.28 |
| 1223 | 13.27±0.66 | 21.44±1.16 | 35.74±0.89 | 873 | 13.13±0.37 | 10.34±1.63 | 23.46±1.67 | 10.96±0.43 | 13.55±1.50 | 27.10±1.24 |
| 1123 | 12.98±0.60 | 20.61±1.79 | 34.42±1.31 | 298 | 8.40±0.73 | 6.97±1.57 | 15.37±1.73 | 8.40±0.73 | 21.89±0.54 | 21.95±1.67 |
| 1023 | 12.20±0.80 | 20.61±0.89 | 32.81±1.95 | − | − | − | − | − | − | − |
| 298 | 15.06±0.312 | 20.61±0.89 | 35.66±0.95 | − | − | − | − | − | − | − |

Sm-Zr (heating) whereas Eu-Zr & Gd-Zr (cooling). The temperature of diffracted volume, Tv was calculated based on the observed difference in melting temperature from cooling trace and *in situ* diffraction.
